# Predictive factors for surgical treatment in preterm neonates with necrotizing enterocolitis: a multicenter case-control study

**DOI:** 10.1007/s00431-020-03892-1

**Published:** 2020-12-02

**Authors:** Sofia el Manouni el Hassani, Hendrik J. Niemarkt, Joep P. M. Derikx, Daniel J. C. Berkhout, Andrea E. Ballón, Margot de Graaf, Willem P. de Boode, Veerle Cossey, Christian V. Hulzebos, Anton H. van Kaam, Boris W. Kramer, Richard A. van Lingen, Daniel C. Vijlbrief, Mirjam M. van Weissenbruch, Marc A. Benninga, Nanne K. H. de Boer, Tim G. J. de Meij

**Affiliations:** 1 Department of Pediatric Gastroenterology, Amsterdam UMC, Academic Medical Center, Amsterdam, the Netherlands; 2 Department of Pediatric Gastroenterology, Amsterdam UMC, VU University Medical Center, Amsterdam, the Netherlands; 3 Neonatal Intensive Care Unit, Máxima Medical Center, Veldhoven, the Netherlands; 4 Department of Pediatric Surgery, Emma Children’s Hospital, Amsterdam UMC, University of Amsterdam and Vrije Universiteit, Amsterdam, the Netherlands; 5 Neonatal Intensive Care Unit, Radboud University Medical Center, Radboud Institute for Health Sciences, Amalia Children’s Hospital, Nijmegen, the Netherlands; 6 Neonatal Intensive Care Unit, University Hospitals Leuven, Leuven, Belgium; 7 Neonatal Intensive Care Unit, Beatrix Children’s Hospital, University Medical Center Groningen, Groningen, the Netherlands; 8 Neonatal Intensive Care Unit, Amsterdam UMC, VU University Medical Center, Amsterdam, the Netherlands; 9 Neonatal Intensive Care Unit, Amsterdam UMC, Academic Medical Center, Amsterdam, the Netherlands; 10 Department of Pediatrics, Maastricht University Medical Center, Maastricht, the Netherlands; 11 Neonatal Intensive Care Unit, Amalia Children’s Center/Isala, Zwolle, the Netherlands; 12 Neonatal Intensive Care Unit, Wilhelmina Children’s Hospital/University Medical Center Utrecht, Utrecht University, Utrecht, the Netherlands; 13 Department of Gastroenterology and Hepatology, Amsterdam Gastroenterology and Metabolism Research Institute, Amsterdam UMC, Vrije Universiteit Amsterdam, Amsterdam, the Netherlands

**Keywords:** Risk factors, Surgery, Prediction, Necrotizing enterocolitis

## Abstract

Necrotizing enterocolitis (NEC) is one of the most common and lethal gastrointestinal diseases in preterm infants. Early recognition of infants in need for surgical intervention might enable early intervention. In this multicenter case-control study, performed in nine neonatal intensive care units, preterm born infants (< 30 weeks of gestation) diagnosed with NEC (stage ≥ IIA) between October 2014 and August 2017 were divided into two groups: (1) medical (conservative treatment) and (2) surgical NEC (sNEC). Perinatal, clinical, and laboratory parameters were collected daily up to clinical onset of NEC. Univariate and multivariate logistic regression analyses were applied to identify potential predictors for sNEC. In total, 73 preterm infants with NEC (41 surgical and 32 medical NEC) were included. A low gestational age (*p* value, adjusted odds ratio [95%CI]; 0.001, 0.91 [0.86–0.96]), no maternal corticosteroid administration (0.025, 0.19 [0.04–0.82]), early onset of NEC (0.003, 0.85 [0.77–0.95]), low serum bicarbonate (0.009, 0.85 [0.76–0.96]), and a hemodynamically significant patent ductus arteriosus for which ibuprofen was administered (0.003, 7.60 [2.03–28.47]) were identified as independent risk factors for sNEC.

*Conclusions*: Our findings may support the clinician to identify infants with increased risk for sNEC, which may facilitate early decisive management and consequently could result in improved prognosis.

**Table:** 

**What is Known:** *• In 27–52% of the infants with NEC, a surgical intervention is indicated during its disease course.* *• Absolute indication for surgical intervention is bowel perforation, whereas fixed bowel loop or clinical deterioration highly suggestive of bowel perforation or necrosi, is a relative indication.*	
**What is New:** *• Lower gestational age, early clinical onset, and no maternal corticosteroids administration are predictors for surgical NEC.* *• Low serum bicarbonate in the 3 days prior clinical onset and patent ductus arteriosus for which ibuprofen was administered predict surgical NEC.*

**Supplementary Information:**

The online version contains supplementary material available at 10.1007/s00431-020-03892-1.

## Introduction

Necrotizing enterocolitis (NEC) is one of the most common gastrointestinal diseases in preterm infants, affecting approximately 7% of very low birth weight infants (VLBW, < 1500 g) and is associated with high mortality rates of 20–30% [1]. Although the pathophysiology of NEC remains to be elucidated, microbiota, genetic predisposition, and immaturity of the gastrointestinal tract are key factors in its etiology [2]. Currently, the diagnosis of NEC is based on a combination of clinical, radiographic, and laboratory parameters. However, timely diagnosis is hampered due to the non-specific nature of clinical symptoms, the absence of specific radiological signs, and the low diagnostic accuracy of laboratory tests [2]. Furthermore, divergent laboratory parameters, such as increased lactate and CRP, commonly occur at an advanced state [3, 4].

In 27–52% of the infants with NEC, a surgical intervention (laparotomy or peritoneal drainage) is indicated during its disease course [5]. The large differences in the percentage of infants requiring surgery might be explained by the differences in study design; in two studies, infants with a birth weight up to 1500 g were included, whereas in one study, infants with a birth weight over 1500 g were included. The study in which infants with higher birth weights were included, a lower percentage of surgical NEC was reported. Absolute indication for surgical intervention is bowel perforation (confirmed by radiographic signs of free gas in the abdomen), whereas fixed bowel loop or clinical deterioration highly suggestive of bowel perforation or necrosis is considered a relative indication [6, 7]. This is mostly based on a combination of radiographic signs and expert opinion, but clinically relevant predictive factors are still limited at best. Furthermore, a high mortality rate of around 30% is observed in infants with NEC-related bowel perforation [7–9]. Apgar score at 1 min, need of inotropic treatment, mean blood pressure, and late-onset sepsis are demonstrated predictors for mortality in surgical NEC [10] .

Previously, studies have aimed to identify predictive clinical parameters of the disease severity in NEC; however, no disease-specific parameters could successfully be identified [11, 12]. Srinivasjois et al. identified a rise of C-reactive protein 72 h after clinical onset, and lactate levels were strong predictors for progression to surgery or death [12]. However, alterations in these values were not significantly different prior clinical onset. Others identified that chorioamnionitis in combination with fetal inflammatory response was more present in infants diagnosed with surgical NEC; however, other studies failed to demonstrate this association [11]. Identification of predictors of disease severity is of importance, since this may facilitate early decisive management such as surgical consultation, additional diagnostics (laboratory investigations or radiographic imaging), transportation to a surgical center, and even surgical intervention. To date, little is known on clinical characteristics and laboratory parameters which could potentially serve as predictors for the clinical deterioration of NEC in preterm infants, ultimately requiring surgical intervention. Therefore, we aimed to identify which perinatal, clinical, and laboratory parameters are associated with increased risk for surgical intervention in premature infants with NEC.

## Material and methods

### Patients and data collection

This cohort study was nested in an ongoing multicenter prospective cohort study, which aims to identify novel biomarkers for early detection of NEC and late-onset sepsis (LOS) [13]. For this study, infants born ≤ 30 weeks of gestation and admitted to one of the nine participating neonatal intensive care units (NICU) situated in the Netherlands and Belgium were eligible to participate. All participating hospitals were in-born centers. For the current study, infants diagnosed with NEC (classified and diagnosed according to the Bell’s criteria) and included between October 2014 and August 2017 were assessed [14]. The local institutional review boards of all nine participating centers granted approval (2014.386 amendment A2016.363). The parents of the included infants gave written informed consent.

Here, the definition of NEC ranged from stage IIA, defined as definite NEC, and stage IIIB, defined as advanced NEC with bowel perforation. Infants with major congenital malformations, isolated spontaneous intestinal perforation (SIP), Bell’s criteria 1 (suspected NEC), and abdominal surgical condition unrelated to NEC were excluded. Infants who needed surgery, but were too ill to undergo surgery, were allocated to the surgical NEC (sNEC) group in order to circumvent selection bias.

In order to identify predictors for the disease course in severe NEC, detailed perinatal, clinical, and laboratory variables are assessed prior to clinical onset of NEC (t0) (Supplemental Table 1). In case of referral to another hospital, daily data collection was ceased; however, details on treatment, surgical procedure, and survival were included in the data collection. All NEC cases were independently reviewed by two experts in the field (HN and TdM), and consensus was met in all cases. NEC cases were allocated to one of the two groups, defined as medical (conservative treatment) or surgical, according to treatment received. Surgery was indicated in infants with evident bowel perforation (pneumoperitoneum) or in infants clinically suspected for having either bowel necrosis or perforation (e.g., no clinical improvement during maximal conservative treatment) but without radiographic confirmation or clinical deterioration despite maximum conservative therapy. Bowel perforation was confirmed by the presence of pneumoperitoneum on an abdominal radiograph or clinical symptoms suspected for bowel perforation and confirmed by either the surgical or histopathological report. Surgical procedures involved peritoneal drainage, exploratory laparotomy with resection of necrosis, primary closure of perforation, and enterostomy with stoma placement.

### Statistical analysis

Statistical Package for the Social Science (SPSS) version 24.0 was used for the statistical analyses. First, frequency distributions and descriptive statistics were retrieved, and the distribution of the data was assessed for normality by applying a Shapiro-Wilk test.

All variables from the medical NEC (mNEC) group were independently compared to the sNEC group using a univariate logistic regression analysis. The results are presented as two-sided *p* values, unadjusted odds ratios (OR), and corresponding 95% confidence intervals (95% CI). A forward selection procedure was performed to create three multivariable logistic prediction models for the dichotomous and continuous dependent variables of sNEC. Considering the small sample size, all variables from the univariate logistic regression with a *p* value of ≤ 0.15 were included. A maximum of three variables per multivariable model were included. Inclusion in the prediction model was set at a *p* value below 0.10 since the sample size of this study was relatively small. Results were presented as adjusted OR (aOR) and corresponding 95% CI.

## Results

**Fig. 1:**
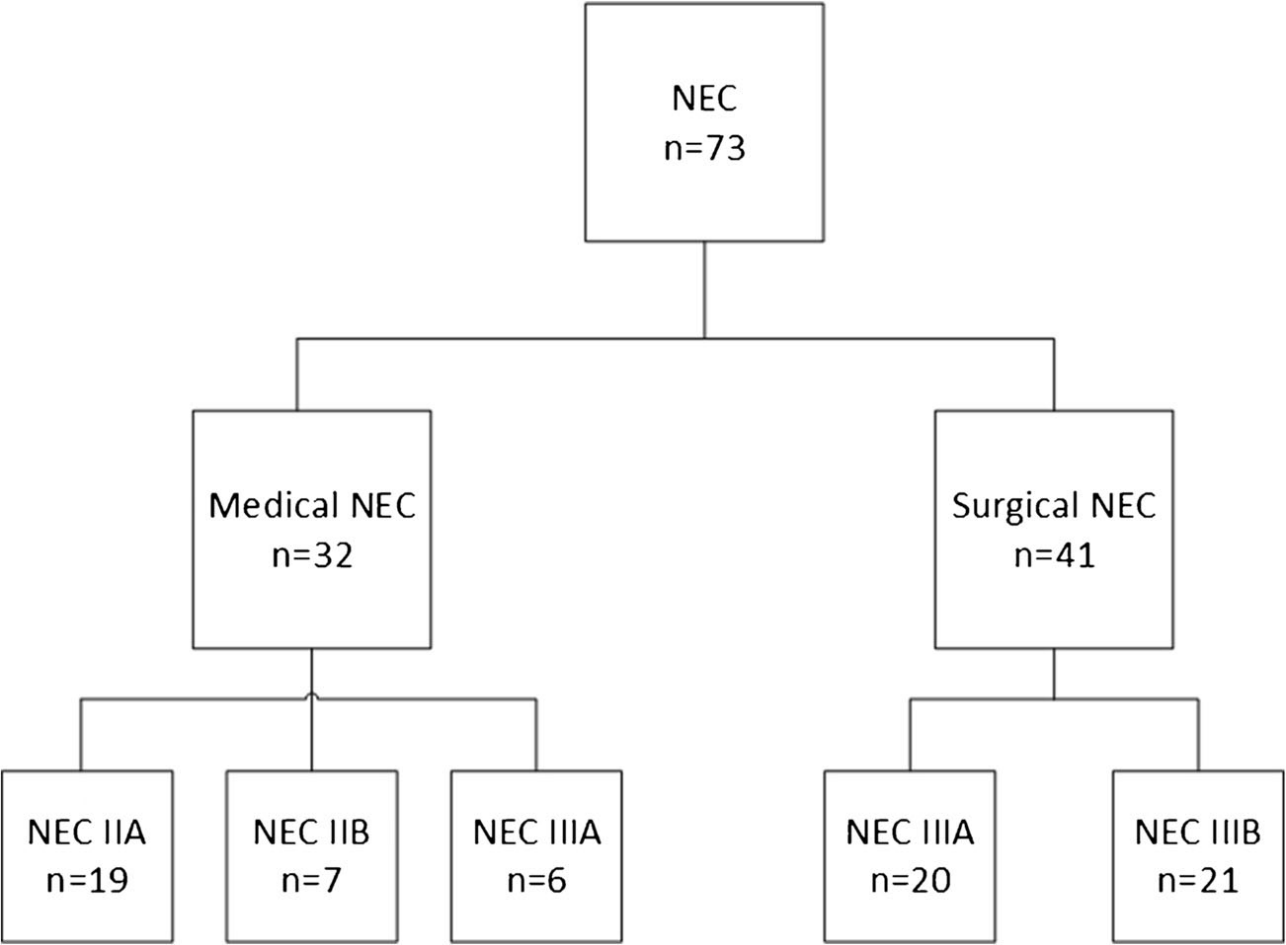
Flowchart of included cases. Here, an overview of the distribution of the groups is provided

In total, 1182 preterm infants were included during the inclusion period, of whom 73 preterm infants (6%) developed NEC stage ≥ IIA and were included for further analyses. Nineteen infants are diagnosed with NEC stage IIA, seven with NEC stage IIB, 26 with NEC stage IIIA, and 21 with NEC stage IIIB (Fig. 1). Postmenstrual age at clinical onset was 27 weeks and 5 days in sNEC group and 29 weeks and 4 days in medical NEC (mNEC) group. Of the included infants, 41 underwent a surgical treatment, and 32 received a non-surgical treatment (Fig. 1). Nine infants had a surgical indication for NEC but were considered clinically too unstable to undergo surgical treatment. These nine patients died as a consequence of NEC. The mortality rate was 11% for NEC IIA (2 out of 19), 0% for NEC IIB (0 out of 7), 65% for NEC IIIA (17 out of 26), and 48% for NEC IIIB (10 out of 21), whereas the mortality rate in mNEC was 9% (3 out of 32) versus 63% (26 out of 41) in sNEC. Mortality rate in the first 120 days of life was significantly higher in the sNEC group, with an odds ratio of 16.76 [95%CI 4.35–64.50], and the median age at death was 16 days in the sNEC group compared to 10 days in the medical group. In Supplemental Table 2, the number of inclusions per center is outlined.

### Perinatal variables

We demonstrated that gestational age (GA) (*p* value, unadjusted OR [95%CI]; 0.001, 0.92 [0.87–0.96]) and birth weight (0.015, 0.997 [0.995–0.999]) were inversely associated with development of sNEC. GA, birth weight and maternal corticosteroid administration were included in the multivariable analysis. We demonstrated that an increase of 1 day in GA, the odds for sNEC decreases by 9%. Maternal corticosteroid administration results in a decrease in odds for sNEC by78% (Table 1).

**Table 1:** Pre- and perinatal characteristics of surgical and medical NEC cases

	Surgical NEC (*n* = 41)	Medical NEC (*n* = 32)	*p* value	Unadjusted OR [95% CI]	*p* value	Adjusted OR [95% CI]
Gestational age (median [IQR], weeks + days)	26 + 1 [24 + 6 – 27 + 3]	28 + 0 [26 + 1–28 + 5]	*0.001*	0.92 [0.87–0.96]	*0.001*	0.91 [0.86–0.96]
Birth weight (mean [SD], g)	871.1 [207.2]	1014.9 [256.8]	*0.015*	0.997 [0.995–0.999]		
Gender male (*n* [%])	22 [53.7]	18 [56.3]	0.825	0.90 [0.36–2.28]		
Delivery mode vaginal delivery (*n* [%])	29 [70.7]	16 [50.0]	0.073	0.41 [0.16–1.09]		
Multiple births (*n* [%])	18 [43.9]	12 [37.5]	0.581	1.30 [0.51–3.36]		
PPROM (*n* [%])	8 [19.5]	3 [9.4]	0.239	2.34 [0.57–9.67]		
Meconium amniotic fluid (*n* [%])	0 [0]	2 [6.3]	0.999	NA		
1-min Apgar (median [IQR])	5 [3-8]	5 [2-6]	0.360	1.10 [0.90–1.34]		
5-min Apgar (median [IQR])	8 [7-9]	7 [6-8]	0.834	1.03 [0.80–1.31]		
Age mother at birth *years* (mean [SD])	31 [4]	31 [6]	0.717	1.02 [0.92–1.12]		
Antibiotics during pregnancy (*n* [%])	8 [19.5]	9 [28.1]	0.390	0.62 [0.21–1.85]		
Antihypertensive medication (*n* [%])	3 [8.6]	6 [21.4]	0.160	0.34 [0.08–1.52]		
Corticosteroids (*n* [%])	30 [73.2]	28 [87.5]	0.141	0.39 [0.11–1.37]	*0.041*	0.22 [0.05–0.94]
Magnesium sulfate (*n* [%])	10 [24.4]	14 [43.8]	0.084	0.42 [0.15–1.13]		
Oxytocin antagonist (*n* [%])	8 [19.5]	9 [28.1]	0.390	0.62 [0.21–1.85]		

*IQR*, interquartile range; *SD*, standard deviation; *n*, number

### Clinical variables

Timing of onset of NEC (*p* value, unadjusted OR [95%CI]; 0.007, 0.90 [0.83–0.97), a hemodynamically significant patent ductus arteriosus (hsPDA) (0.007, 4.14 [1.46–11.69]) for which ibuprofen was administered, not achieving full enteral feeding prior clinical onset (0.030, 0.35[ 0.13–0.90]), and an episode of LOS within 72 h prior clinical onset of NEC (0.022, 3.11 [1.18–8.21]) are associated with the development of sNEC (Table 2). Day of life at clinical onset of NEC, hemodynamically significant PDA for which ibuprofen administration and an episode of LOS within 72 h prior clinical onset of NEC were included in the multivariable analysis. Early onset of NEC, defined as every postnatal day earlier NEC was diagnosed, increases the odds for sNEC by 15%, whereas a hsPDA for which ibuprofen was administered increases the odds by660%.

**Table 2:** Clinical features for surgical and medical NEC in the period preceding diagnosis (*T*_0_)

	Surgical NEC (*n* = 41)	Medical NEC (*n* = 32)	*p* value	Unadjusted OR [95% CI]	*p* value	Adjusted OR [95% CI]
NEC onset days (median [IQR])	10 [7–15]	15 [9–21]	*0.007*	0.90 [0.83–0.97]	*0.003*	0.85 [0.77-0.95]
Time until first defecation days (median [IQR])	2 [1–3]	2 [1–4]	0.716	0.95 [0.72–1.26]		
Late onset sepsis < 72 h prior *t*0 (*n* [%])	24 [58.5]	10 [31.3]	*0.022*	3.11 [1.18–8.21]		
Hemodynamically significant PDA (*n* [%])	22 [53.7]	7 [21.9]	*0.007*	4.14 [1.46–11.69]	*0.003*	7.60 [2.03-28.47]
RBC transfusion days (median [IQR])	1 [1–2]	1 [0–3]	0.619	0.92 [0.67–1.27]		
Time between last dose and t0 days (median [IQR])	2 [1–8]	2 [1–6]	0.901	0.99 [0.88–1.12]		
Mechanic ventilation exposure (*n* [%])	30 [73.2]	18 [56.3]	0.134	2.12 [0.79–5.67]		
Mechanic ventilation days (median [IQR])	4 [1-6]	3 [0–9]	0.767	1.01 [0.92–1.11]		
Enteral feeding type (*n* [%])						
Breast milk	14 [41.2]	12 [41.4]	0.346	*Reference*		
Formula milk	5 [12.2]	1 [3.14]	0.211	4.29 [0.44–41.95]		
Combination	15 [44.1]	16 [55.2]	0.682	0.80 [0.28–2.28]		
Increase enteral feeding (mean [SD]) mL/kg/day	11.14 [4.87]	11.08 [3.20]	0.949	1.00 [0.90–1.12]		
Achieved full enteral feeding (*n* [%])	15 [36.6]	20 [62.5]	*0.030*	0.35 [0.13–0.90]		
Age at full enteral feeding in days (median [IQR])	9 [6–12]	10 [9–12]	0.227	0.86 [0.67–1.10]		
Total days parenteral feeding (median [IQR])	9 [5–13]	9 [5–12]	0.894	1.01 [0.90–1.13]		
Postpartum antibiotics (*n* [%])						
Not administered	3 [7.3]	9 [28.1]	0.081	*Reference*		
1–3 days administered	22 [53.7]	14 [43.8]	0.038	4.71 [1.09–20.47]		
> 3 days administered	16 [39.0]	9 [28.1]	0.033	5.33 [1.14–24.90]		
Antibiotics days (median (IQR]) Time between last dose and t0 (median [IQR]) days	5 [3–9] 1 [0–5]	6 [3–8] 2 [0–7]	0.844 0.115	0.99 [0.89–1.11] 0.92 [0.82–1.02]		
Antibiotic exposure per group (*n* [%])						
Aminoglycoside	34 [82.9]	24 [75.0]	0.408	1.62 [0.52–5.07]		
Carbapenem	2 [4.9]	2 [6.3]	0.799	0.77 [0.10–5.78]		
Cephalosporin	12 [29.3]	4 [12.5]	0.094	2.90 [0.83–10.06]		
Glycopeptide	9 [22.0]	5 [15.6]	0.497	1.52 [0.45–5.08]		
Penicillin (-clavulanic acid)	39 [95.1]	28 [87.5]	0.255	2.79 [0.48–16.28]		
Medication (*n* [%])						
Antimycotics	9 [22.0]	6 [18.8]	0.737	1.22 [0.38–3.87]		
Corticosteroids	5 [12.2]	3 [9.4]	0.703	1.34 [0.30–6.09]		
Inotropes	15 [36.6]	6 [18.8]	0.100	2.50 [0.84–7.45]		

*IQR*, interquartile range; *n*, number; *RBC*, red blood cell; *PDA*, patent ductus arteriosus; *t0*, clinical onset of NEC

### Laboratory variables

In Table 3, the laboratory variables (defined as most deviating value in the 3 days prior clinical onset) for both subgroups are displayed. Low serum bicarbonate (*p* value, unadjusted OR [95% CI]; 0.003, 0.83 [0.74–0.94]), high base deficit (0.004, 0.85 [0.76–0.95]), and low platelets counts (0.019, 0.995 [0.990–0.998]) were associated with increased risk for sNEC. After including these variables in the multivariable analysis, a drop of one point in serum bicarbonate increases the odds for sNEC by 14%.

**Table 3:** Laboratory values prior clinical onset (*t*0)

	Surgical NEC (*n* = 41)	Medical NEC (*n* = 32)	*p* value	Unadjusted OR [95% CI]	*p* value	Adjusted OR
Hemoglobin mmol/L (mean [SD])	8.0 [1.06]	8.1 [1.7]	0.656	0.92 [0.62–1.35]		
C-reactive protein mg/L (median [IQR])	10 [2–57]	5 [2–18]	0.184	1.02 [0.99–1.04]		
Arterial blood gas mean [SD]						
pH pCO_2_ kPa	7.19 [0.10] 7.61 [1.85]	7.25 [0.11] 8.02 [3.06]	0.058 0.562	0.00 [0.00–1.23] 0.93 [0.73–1.18]		
pO_2_ kPa	4.93 [1.93]	4.25 [3.60]	0.734	0.96 [0.76–1.22]		
HCO_3-_mmol/L	20.35 [4.92]	26.71 [6.84]	*0.003*	0.83 [0.74–0.94]	*0.043*	0.863 [0.749–0.995]
Base excess mmol/L	− 8.0 [5.02]	− 1.6 [7.2]	*0.004*	0.85 [0.76–0.95]		
Lactate mmol/L	1.92 [0.71]	2.73 [1.39]	0.145	0.42 [0.13–1.35]		
Platelet counts × 10^9^/L (median [IQR])	144 [93–262]	270 [179–388]	*0.019*	0.995 [0.990–0.999]		
Leucocytes × 10^9^/L (median [IQR])	19.4 [10.6–29.0]	16.2 [10.1–32.7]	0.945	0.998 [0.952–1.047]		

*SD*, standard deviation; *NEC*, necrotizing enterocolitis; *OR*, odds ratio; *IQR*, interquartile range; *HCO*_3-_, bicarbonate

## Discussion

In the current study, perinatal, clinical, and laboratory variables were prospectively collected before NEC onset and subsequently compared between infants undergoing either medical or surgical treatment. Lower GA, no maternal corticosteroid administration, early clinical onset of NEC, a low bicarbonate, and hsPDA for which ibuprofen was administered were identified as independent risk factors for sNEC.

### Perinatal variables

Antenatal corticosteroid treatment was associated with lower risk of sNEC. Several studies have assessed the (side) effects of antenatal exposure to glucocorticoids. In a large multicenter prospective cohort study, it was demonstrated that infants exposed to antenatal corticosteroids were less likely to develop NEC stage 2 or higher [15]. It has been proposed that glucocorticosteroids play an important role in gut maturation. Studies in rats have indicated that glucocorticoids induce the activity of lactase, sucrase, and fucosylation and reduce the activity of sialylation, which are indicative of intestinal maturation [16]. Furthermore, antenatal steroids are associated with a more stable blood pressure and improved lung maturation, essential for adequate intestinal perfusion and oxygenation. Moreover, antenatal steroids are associated with a decrease in incidence of PDA, since these attenuate the sensitivity of the DA to PGE_2_ and increase the PGE_2_ catabolism.

Robust evidence exists on the presence of an association between low GA and development of NEC [17]. In the current study, we identified GA as an independent predictor for the development of sNEC. This can be explained by immaturity of the gut in lower GA, contributing to increased vulnerability for bowel perforation. Extremely preterm born infants are also more likely to suffer from comorbidities which influence the natural course of NEC, such as infectious diseases and PDA. Furthermore, immaturity of the immune system has been also associated with an excessive inflammatory response, which tends to play a key role in the inflammatory response in NEC pathogenesis and might consequently provoke gut perforation [18].

### Clinical characteristics

In the current study, early clinical onset of NEC was identified as an independent predictor for development of sNEC. This is in accordance with the findings of Duci et al., who demonstrated that infants requiring surgical treatment for NEC were diagnosed with NEC at a lower postnatal age, compared to infants receiving conservative medical treatment [19]. By increasing postnatal age, maturation of the intestines occurs, which may reduce the risk for severe NEC ultimately necessitating surgery. Saleem et al. demonstrated that maturation of the intestinal barrier in preterm infants is GA and postnatal age dependent, thus reducing the risk for the development of advanced NEC [20].

Not achieving full enteral feeding prior clinical onset and late-onset sepsis within 72 h prior clinical onset was associated with the development of sNEC. As mentioned above, early clinical onset was identified as an independent risk factor for sNEC, since these children are younger; it is more likely that they did not achieve full enteral feeding due to their postnatal age. In a previous study, it has been demonstrated that LOS occurs concurrently with NEC (within 72 h preceding clinical onset) in 43.7% of the NEC cases, resulting in higher odds (aOR [95%CI]; 3.51 [1.98–6.24]) for sNEC compared to NEC cases without LOS [21]. In the current study, these variables were not identified as independent predictors for sNEC.

Conflicting results have been reported for the influence of a PDA on the natural course of NEC. It has been described that NEC cases with PDA were associated with a better outcome compared to NEC patients without PDA [22]. Authors hypothesized that a NEC-like presentation in infants with a PDA might be a consequence of decreased mesenteric perfusion, retrograde diastolic flow, and low diastolic pressure resulting from PDA, rather than as a consequence of gut immaturity, as is seen in classic NEC cases, and, is therefore, associated with a better clinical outcome. In another study, it was reported that PDA in NEC patients was associated with increased mortality rates; however, in that study, infants with suspected NEC (stage 1) were also included, which could have resulted in bias in study outcome [23]. Kessler et al. demonstrated an association between concurrent diagnosis of NEC and PDA and also an increased mortality [24]. However, the authors did not look into the attributed risk for surgical intervention in infants concurrently diagnosed with NEC and PDA. In the current study, majority of the infants treated for a hsPDA received ibuprofen, which was found to be associated with the development of sNEC. In a meta-analysis in which the classical medical treatment of a hsPDA, indomethacin, was compared to ibuprofen, it was demonstrated that there was no increased risk for the development of NEC [25]. We hypothesize that the increased risk for clinical deterioration of NEC in presence of a hsPDA could be attributed to a decreased intestinal perfusion and decreased oxygenation resulting from PDA. Also, a hsPDA is inversely associated with GA [26]. The question remains whether the presence or the treatment of a PDA is causally related to NEC that must be surgically treated or that the higher incidence of PDA in the surgical group should be considered as a marker of immaturity.

### Laboratory values

Thrombocytopenia, defined as a platelet count < 150 × 10^9^/L, has previously been described to be associated with a poor clinical course of NEC [27]. In the current study, a median platelet count of 144 × 10^9^/L and 270 × 10^9^/L was found for sNEC and mNEC, respectively. In the current study, an association between lower platelet counts and the development of sNEC was identified. However, low platelet count was not identified as an independent predictor for sNEC.

In our cohort, we demonstrated an association between a low serum HCO_3_− and large negative base excess preceding clinical onset of NEC and the development of sNEC. Low serum bicarbonate can be the result of metabolic acidosis in infants with poor circulation or by abdominal leakage of bicarbonate in severe cases. It is hardly surprising that these values are more deviant in the surgical group, since both serum HCO_3_- and base excess reflect the metabolic status of the infant. It has been reported that infants requiring surgical intervention are more likely to present with divergent objective metrics of metabolic derangement, such as low serum HCO_3_− and a high base excess [28]. We identified a low serum HCO_3_− as an independent risk factor for sNEC, whereas a large negative base excess preceding clinical onset of NEC was associated with sNEC.

One of the strengths of this study is the prospective multicenter study design, which allowed for the inclusion of a wide variety of perinatal, clinical, and laboratory information collected daily, allowing for detailed evaluation of possible factors predictive for the clinical course of NEC. The current study was limited by the relatively small sample size of NEC cases, therefore, limiting the number of variables that could be added into the multivariable model. Consequently, three separate models were constructed allowing the inclusion of all variables of interest into the multivariable models. Also, due to the small sample size, no correction for center variation could be performed.

In conclusion, we identified low GA, no maternal corticosteroids administration, early onset of NEC, hsPDA for which ibuprofen was administered and low bicarbonate as independent predictors for surgery in preterm infants < 30 weeks with NEC. Our findings may support the clinician to identify infants with increased risk for sNEC, potentially leading to earlier surgical consultation, additional diagnostics, or even surgical intervention, which consequently may lead to an improved outcome.

## Supplementary Information

ESM 1 (DOCX 13 kb)

ESM 2 (DOCX 12 kb)

## Data Availability

N/A

## Abbreviations

GA: Gestational age
hsPDA: Hemodynamically significant patent ductus arteriosus
LOS: Late-onset sepsis
mNEC: Medical necrotizing enterocolitis
NEC: Necrotizing enterocolitis
NICU: Neonatal intensive care unit
OR: Odds ratio
sNEC: Surgical necrotizing enterocolitis
SIP: Spontaneous intestinal perforation
