# Supplementary material for: Predictive factors for surgical treatment in preterm neonates with necrotizing enterocolitis: a multicenter case-control study
Source: Eur J Pediatr. 2020 Dec 2;180(2):617–25. doi: 10.1007/s00431-020-03892-1 (PMC7813726; doi:10.1007/s00431-020-03892-1)
Supplement: Supplementary file 1 — (DOCX 13 kb) [file 431_2020_3892_MOESM1_ESM.docx]

| **Supplemental Table 1.** Variables assessed and corresponding definitions | |
| --- | --- |
| **Demographical variables** | **Definitions** |
| Gestational age |  |
| Birthweight |  |
| Gender |  |
| Delivery mode |  |
| Multiple birth |  |
| Apgar score |  |
| Premature rupture of membranes | Rupture of membranes >24 hours prior delivery. |
| **Clinical variables** | |
| Patent Ductus Arteriosus | Hemodynamically significant PDA, defined as medical treatment with ibuprofen. |
| NEC onset | Day of life on which the symptoms of NEC started |
| Late onset sepsis | Episode of infection >72 hours after birth and within 72 hours prior NEC onset, meeting Vermont Oxford criteria for sepsis:  (1) Clinical symptoms of systemic infection  (2) Isolation of a pathogen from blood culture  (3) Antibiotic treatment of ≥ 5 days targeting the causative pathogen. |
| Antibiotics | (1) Cumulative number of days antibiotics administered  (2) Number of days antibiotics administered directly post-partum: (a) 0 days, (b) ≤3 days, (c) > 3 days. |
| Mechanical ventilation | Ventilation requiring intubation. Total cumulative number of days were noted. |
| Erythrocytes transfusion | Total cumulative number of days a RBC transfusion was administered. |
| Parenteral feeding | Cumulative number of days any nutritional formula was parenterally administered |
| Enteral feeding practice | (1) breast milk fed, daily > 80% breast milk, including donor milk  (2) formula fed, daily > 50% formula milk  (3) combination of breast and formula milk.  Full enteral feeding: two consecutive days without parenteral feeding or enteral feeding volume of 120 mL/kg/day.  Increase or decrease of daily feeding volume prior NEC onset was noted relative to the neonates birth weight (mL/kg/day). |
| Laboratory tests | Most deviating value of white blood cell count, hemoglobin, platelet count, CRP and arterial blood gas parameters, within three days prior clinical onset. |
| Mortality | death from any cause in the first 120 days of life. |

**Abbreviations** PDA, patent ductus arteriosus; NEC, necrotizing enterocolitis; RBC, red blood cells
