# Supplementary material for: Predictive factors for surgical treatment in preterm neonates with necrotizing enterocolitis: a multicenter case-control study
Source: Eur J Pediatr. 2020 Dec 2;180(2):617–25. doi: 10.1007/s00431-020-03892-1 (PMC7813726; doi:10.1007/s00431-020-03892-1)
Supplement: Supplementary file 2 — (DOCX 12 kb) [file 431_2020_3892_MOESM2_ESM.docx]

| Supplemental Table 2. Number of inclusions per participating center | | |
| --- | --- | --- |
|  | Surgical NEC (n=41)  (n[%]) | Medical NEC (n=32)  (n[%]) |
| 1 | 5 [12.2] | 9 [28.1] |
| 2 | 3 [7.3] | 8 [25.0] |
| 3 | 7 [17.1] | 3 [9.4] |
| 4 | 3 [7.3] | 2 [6.3] |
| 5 | 2 [4.9] | 3 [9.4] |
| 6 | 1 [2.4] | 0 [0.0] |
| 7 | 6 [14.6] | 3 [9.4] |
| 8 | 5 [12.2] | 3 [9.4] |
| 9 | 9 [22.0] | 1 [3.1] |
